# Emerging role of the cGAS-STING pathway in cardiovascular diseases: biologic function, mechanisms and targeted therapy

**DOI:** 10.1186/s10020-025-01273-8

**Published:** 2025-06-04

**Authors:** Junke Mou, Yuwu Chen, Xinxin Zhu, Biyi Xu, Mengyang Wang, Jiani Xie, Tong Lin, Qishuo Gu, Qiuwen Wu, Ziao Che, Ji Li, Xing Luo, Haibo Jia, Bo Yu

**Affiliations:** 1 Department of Cardiology, National Key Laboratory of Frigid Zone Cardiovascular Diseases, 2nd Affiliated Hospital of Harbin Medical University, Harbin, 150001 PR China; 2 The Key Laboratory of Myocardial Ischemia, Chinese Ministry of Education, Harbin, 150001 PR China

**Keywords:** Mitochondrial DNA, cGAS/STING, Cardiovascular diseases, Redox balance

## Abstract

Currently, cardiovascular diseases (CVDs) represent a substantial threat to human health and wellness. Accumulating evidence has increasingly highlighted the pivotal role of the DNA-activated cyclic GMP-AMP (cGAMP) synthase (cGAS)-stimulator of interferon gene (STING) pathway in the progression of CVDs. The enhanced type I interferon response and the release of pro-inflammatory mediators disrupt cellular redox balance and trigger various cell death modalities, potentially leading to the emergence of adverse events such as atherosclerosis. Importantly, pharmacological or genetic suppression of the cGAS-STING axis has shown promise in alleviating cardiac injury symptoms. Moderate activation of STING can elicit effective immune responses against myocardial virus infection. This review elucidates the biochemical properties of cGAS and STING, as well as the mechanistic insights into the cGAS-STING pathway within the cardiovascular system, emphasizing the therapeutic potential of cGAS-STING modulators for CVD management.

## Introduction

Cardiovascular diseases (CVDs) encompass a range of conditions, including coronary heart disease, heart failure (HF) and other cardiac disorders. Despite the continuous advancement of medical technology and the clinical application of therapeutics such as statins, the morbidity and mortality rates of CVDs remain high (Goff et al. 2021). Consequently, it is imperative to identify novel therapeutic targets aimed at preventing the onset and progression of CVDs, as well as improving patient outcomes.

Cyclic GMP-AMP (cGAMP) synthase (cGAS) is a cytoplasmic double-stranded DNA (dsDNA) sensor that initiates the synthesis of cGAMP upon binding to dsDNA (Kato et al. 2017; Shu et al. 2014). Subsequently, cGAMP further activates the stimulator of interferon gene (STING) and its downstream signaling cascades, such as the cGAS-STING-TNAK-binding kinase 1 (TBK1)-Interferon regulatory factor 3 (IRF3) pathway and the cGAS-STING-nuclear factor kappa-B (NF-κB) pathway (Chen et al. 2016; Zhang et al. 2020). IRF3 facilitates the synthesis of type I interferon (IFN-1), which is crucial in infectious diseases, sterile inflammation, tumors, and autoimmune diseases (Cañadas et al. 2018; Kumari et al. 2016; Yum et al., 2021). NF-κB has been recognized for its role as a primary pro-inflammatory signaling molecule that modulates cellular reactions to environmental stimuli (Lawrence 2009; Wibisana et al., 2022).

Extensive studies have revealed that the activation of cGAS-STING pathway is implicated in the pathophysiology of CVDs, including atherosclerosis, myocardial infarction (MI), ischemia-reperfusion (I/R) injury, myocarditis, cardiomyopathy, HF, and aortic aneurysm and dissection (AAD) (Liu et al. 2023b; Luo et al. 2020; Ma et al. 2023; Pham et al. 2021; Qin et al., 2023; Wang et al. 2024a; Zhai et al. 2024). Specifically, pathological scenarios like mitochondrial damage or cell death in cardiomyocytes, endothelial cells, macrophages or smooth muscle cells (SMCs) result in the release of mitochondrial or nuclear DNA into the cytoplasm, which subsequently activates the cGAS-STING pathway (An et al. 2023; Fang et al. 2023; Luo et al. 2020; Pham et al. 2021). The enhanced IFN-1 response and the secretion of pro-inflammatory mediators lead to chronic inflammatory responses and disturbances in critical intracellular homeostasis mechanisms, promoting cellular senescence, apoptosis, and pyroptosis, ultimately resulting in cardiac structural and functional abnormalities (Bi et al. 2021; Huangfu et al. 2021; Liu et al. 2023b; Xie et al., 2023). Notably, positive outcomes linked to STING activation have been reported in certain studies, including the reversal of autophagy activation in cardiomyocytes and fibroblasts, as well as the suppression of myocardial viral infections (Song et al. 2019; Xiong et al. 2021). Silencing of cGAS and STING genes, along with the application of inhibitors, has been demonstrated to exert cardioprotective effects (Guo et al. 2023; Hua et al. 2023; Li et al. 2023a). Additionally, vaccines adjuvated with STING agonists have shown promising immune responses against myocardial virus infection (Zhang et al., 2024). These findings imply that targeting the cGAS-STING pathway could represent a viable therapeutic avenue for CVD management. In this review, we discuss the biochemical properties of cGAS and STING, while selectively summarizing relevant literature regarding the implications of the cGAS-STING pathway in CVDs. We also focus on the potential roles of cGAS-STING inhibitors or agonists for cardiovascular diseases therapy, in order to provide useful insights into the precise treatment of CVDs.

## cGAS overview

### The structure of cGAS

**Fig. 1:**
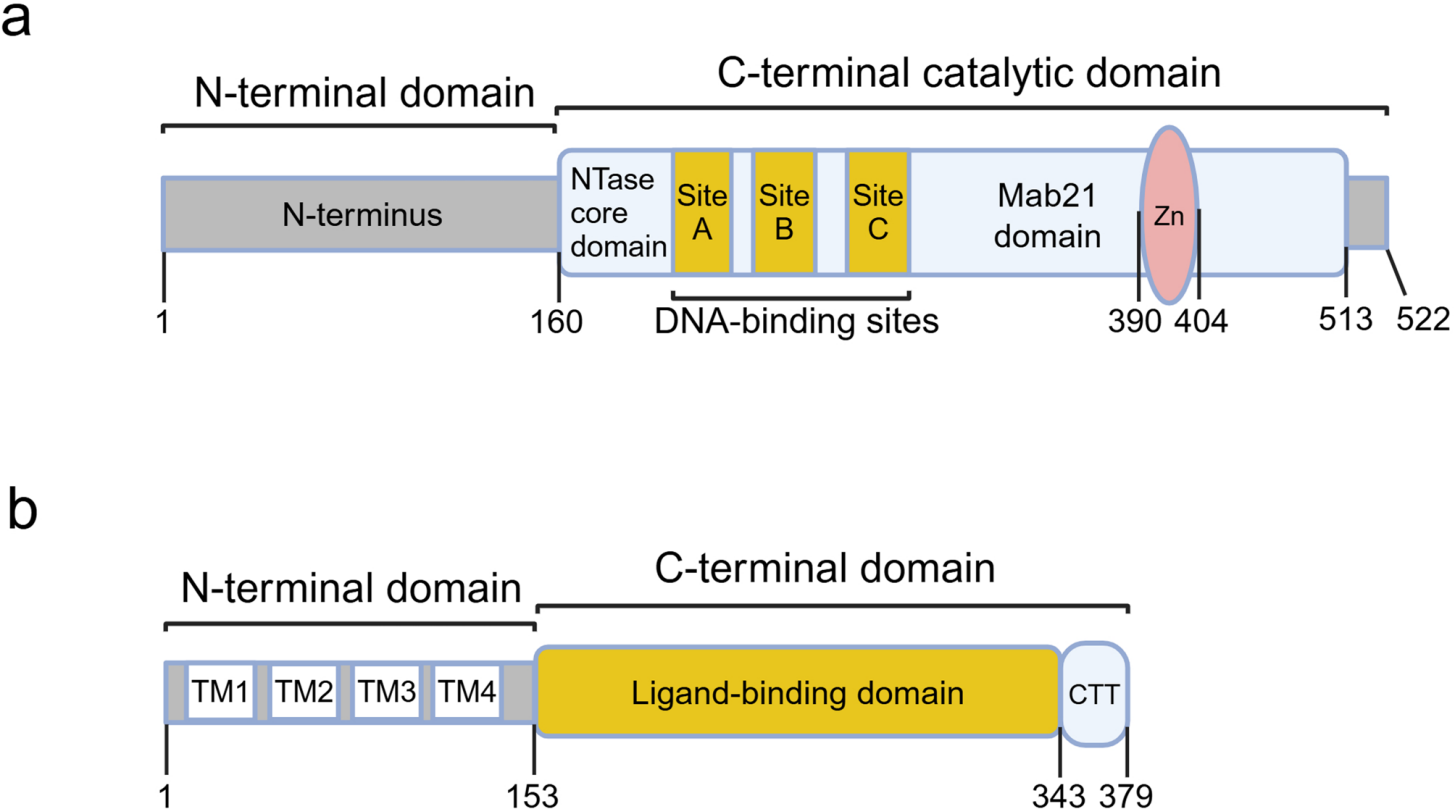
Molecular structure of cGAS and STING. **a** A diagram of the domain organization of human cGAS. **b** A diagram of the domain organization of human STING. *NTase*, nucleotidyltransferase; *TM*, transmembrane; *CTT*, C-terminal tail

cGAS is a 522-amino acid protein of the nucleotidyltransferase (NTase) family with a molecular weight of 60 kDa. Its structure comprises a disordered N-terminal domain and a conserved C-terminal catalytic domain (Gentili et al. 2019; Zhang et al. 2020). The C-terminal catalytic structural domain is primarily composed of a NTase core structural domain and a Mab21 structural domain, presenting a bilobed configuration. The N-terminal lobe exhibits the classical NTase fold, while the C-terminal lobe is characterized by a tightly packed helical bundle that contains a zinc-finger structure (Wu et al. 2014). The primary substrate binding site (A site) is formed by the deep groove edge between the two lobes (Li et al. 2013). The adjacent secondary site (B site) is also involved in interactions with DNA (Zhang et al. 2014). Additionally, a recently identified site (C site) has been implicated in the activation of cGAS (Xie et al. 2019) (Fig. 1a).

### cGAS in the cytoplasm: DNA recognition and cGAMP production

**Fig. 2:**
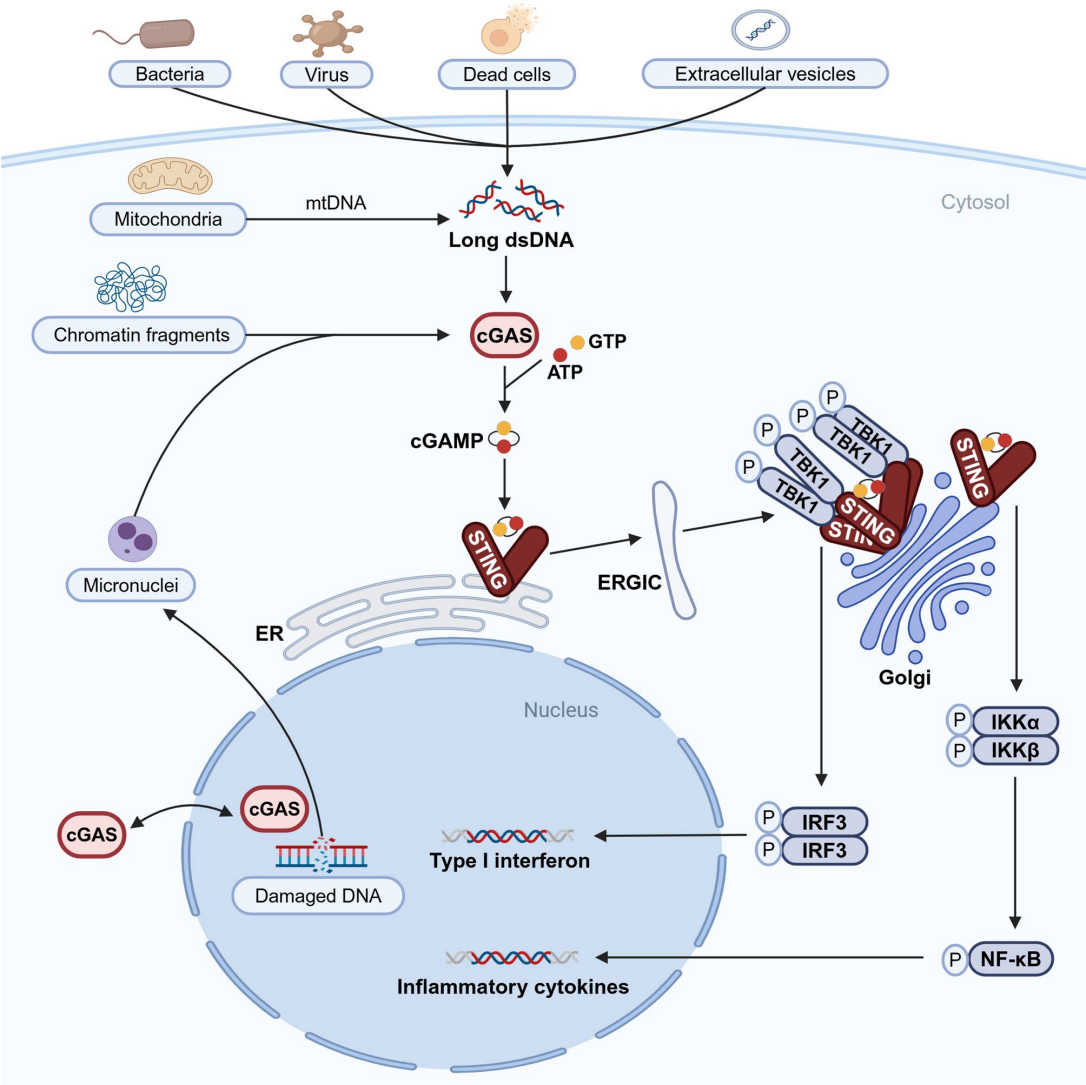
Molecular mechanism of cGAS-STING signaling. Nucleo-cytoplasmic transport of cGAS ensures that cGAS performs its function in the proper localization. Nuclear cGAS monitors and transports damaged DNA. cGAS in the cytoplasm functions as a DNA sensor. Upon recognition of exogenous dsDNA or endogenous dsDNA, cGAS-dsDNA complexes catalyse ATP and GTP to generate cGAMP. Activated STING recruits and phosphorylates TBK1 and IKK. TBK1 further recruits and phosphorylates IRF3, which forms a dimer that enters the nucleus and induces the production of type I interferon. IKK recruits and phosphorylates NF-κB. The activated NF-κB moved to the nucleus and initiated the transcription of downstream inflammatory cytokines. *cGAMP*, cyclic GMP-AMP; *cGAS*, cyclic GMP-AMP synthase; *STING*, stimulator of interferon gene; *dsDNA*, double-stranded DNA; *ATP*, adenosine triphosphate; *GTP*, guanosine triphosphate; *ER*, endoplasmic reticulum; *ERGIC*, ER–Golgi intermediate compartment; *TBK1*, TANK-binding kinase 1; *IRF3*, interferon regulatory factor 3; *IKK*, IκB kinase; *NF-κB*, nuclear factor kappa-B

cGAS is primarily localized in the cytoplasm and the nucleus (Liu et al. 2018; Lu et al. 2024b; Zhao et al. 2020; Zhong et al. 2020). Within the cytoplasm, cGAS functions as a DNA sensor, recognizing dsDNA from both exogenous and endogenous sources (Sun et al. 2013). Exogenous dsDNA can originate from various entities, including microorganisms, virus, dead cells, and extracellular vesicles. Endogenous dsDNA is derived from DNA released from mitochondria, chromatin fragments and damaged DNA packaged into micronuclei in the nucleus (Hopfner et al., 2020; Mackenzie et al. 2017; Oduro et al. 2022) (Fig. 2).

The capacity of cGAS to recognize dsDNA is independent of the nucleotide sequence but is determined by the structural characteristics of the sugar-phosphate backbone (Chen et al. 2016; Zhang et al. 2014). This nonspecific and broad recognition capability expands the scope of the immune response mediated by cGAS. Additionally, the interaction of cGAS with dsDNA exhibits a dependence on the length of the dsDNA. It has been demonstrated that cGAS activation at low DNA concentrations is efficient in long length dsDNA and is not effectively achieved in short length dsDNA (Luecke et al. 2017). The feature protects cells against spurious activation of cGAS on limited and short dsDNA (Decout et al. 2021). Following the non-specific binding of cGAS to the sugar-phosphate backbone of long dsDNA, two cGAS monomers sandwich two dsDNA strands. Site A of one cGAS monomer binds one dsDNA strand, while site B of another monomer binds another dsDNA strand, resulting in a 2:2 cGAS-dsDNA complex. Subsequently, the complex utilizes adenosine triphosphate (ATP) and guanosine triphosphate (GTP) as substrates to synthesize the 2′3′ cGAMP, which features two phosphodiester linkages (Hopfner et al., 2020; Li et al. 2013; Wu et al. 2014) (Fig. 2).

### cGAS in the nucleus: functions independent of DNA activation

In the nucleus, cGAS is not activated by its own DNA, which depends on the regulation by nucleosomes and non-nucleosomes. Specifically, cGAS exhibits a greater affinity for recombinant nucleosomes compared to DNA, binding to negatively charged plaques formed by the nucleosome histones H2 A and H2B via its B site, which spatially inhibits its dimerization process (Boyer et al. 2020; Cao et al. 2020; Michalski et al. 2020; Zhao et al. 2020; Zierhut et al. 2019). Additionally, the hyperphosphorylation of N-terminal structural domain of cGAS and competitive binding of the barrier-to-autointegration factor to DNA further restrict its activity within the nuclear environment (Guey et al. 2020; Li et al., 2021).

Despite these constraints, cGAS is capable of undertaking supplementary roles within the nucleus. In instances of DNA damage and chromatin instability, nuclear cGAS facilitates the translocation of these damaged structures as micronuclei into the cytoplasm, subsequently activating cGAS (Mackenzie et al. 2017). Furthermore, upon cytoplasmic stimulation by DNA, nuclear cGAS replenishes the cytoplasmic reservoir of cGAS via the CRM1-mediated nuclear export pathway (Sun et al. 2021). In response to nuclear DNA damage, cGAS is transported from the cytoplasm to the nucleus through an input protein α-dependent mechanism (Liu et al. 2018) (Fig. 2).

## STING overview

STING is a 379-amino acid protein (Shang et al. 2012). Its structure has been elucidated through cryo-electron microscopy, revealing that it functions as a dimer in the cytoplasm (Shang et al. 2019). Each STING molecule features an N-terminal structural domain and a globular C-terminal structural domain (CTD) (Shang et al. 2012; Wu et al. 2014). The N-terminal domain predominantly comprises four transmembrane (TM) segments that facilitate the anchoring of STING to the endoplasmic reticulum (ER) membrane. The CTD includes a cytoplasmic ligand-binding domain (LBD) and a C-terminal tail (CTT) (Wu et al. 2014) (Fig. 1b). In its dimeric form, the two LBDs create a ‘V’-shaped ligand-binding pocket. Upon binding to cGAMP, the LBDs rotate 180° relative to the TM domains (Shang et al. 2019). This conformational change results in the closing of the ligand-binding pocket and the formation of a disulfide-linked polymer via cysteine residues (Ergun et al. 2019). The activated polymer is subsequently translocated along the ER to the ER-Golgi intermediate compartment (ERGIC) and then to the Golgi apparatus, where the cysteine residues undergo palmitoylation, thereby activating downstream signaling, specifically the cGAS-STING-TBK1-IRF3 pathway and the cGAS-STING-NF-κB pathway (Dobbs et al. 2015; Mukai et al. 2016; Oduro et al. 2022; Srikanth et al. 2019) (Fig. 2).

## Mitochondrial DNA: a critical initiator of the cGAS-STING pathway

Mitochondrial DNA (mtDNA) is a circular, double-stranded molecule situated within the mitochondrial matrix, responsible for encoding critical subunits of the oxidative phosphorylation pathway (Anderson et al. 1981; Chung et al. 2022). The mitochondria in living organisms produces substantial energy, with mtDNA positioned centrally in the energy generation process, thus experiencing persistent oxidative stress (Sharma et al. 2019). While the encapsulation by mitochondrial transcription factor A (TFAM) may provide a degree of shielding against damage caused by reactive oxygen species generated during respiration and pathological metabolic events, mtDNA is still devoid of robust protective and repair means (Kunkel et al. 2016; Song et al. 2024). The mechanisms and rationale behind mtDNA leakage from mitochondria remain inadequately elucidated. It has been proposed that in the absence of TFAM, the abnormal packaging of mtDNA facilitates its release into the cytosol, where it is recognized by the DNA sensor cGAS, subsequently activating STING-IRF3-mediated pathways of innate immune signaling (West et al., 2017). Evidence indicates that apoptotic mechanisms governed by BAX and BAK can facilitate the release of mtDNA, which then triggers cGAS-STING signaling pathway (Rongvaux et al. 2014; White et al. 2014). Furthermore, impairment of the mitochondrial autophagy pathway may lead to mtDNA fragmentation and increased release, which is one of the reasons for cytoplasmic mtDNA accumulation (Chao et al. 2021; Yan et al., 2019).

## The cGAS-STING pathway modulates inflammation, cellular events, and metabolism

### The cGAS-STING pathway and inflammation

Inflammation constitutes a protective physiological response elicited by the body in reaction to detrimental stimuli, such as pathogens, physical trauma, or toxic agents (Medzhitov 2008). The interplay between interferon (IFN) signaling pathways and viral countermeasures constitutes a highly intricate network (Hoffmann et al. 2015). Zhang et al. have elucidated the structural framework of the cGAS-STING-TBK1-IRF3 classical pathway responsible for the secretion of IFN-1. Following the activation by cGAMP binding, the STING dimer initially recruits the TBK1 dimer through the TBK1 binding motif situated at the CTT. Subsequently, within large oligomeric complexes, TBK1 phosphorylates the Ser366 residue in the adjacent STING tail, facilitating the recruitment of IRF3. TBK1 further phosphorylates IRF3 (Zhang et al. 2019a). Ultimately, the phosphorylated IRF3 forms a dimer that migrates into the nucleus, triggering the expression of IFN-1 (Liu et al. 2015) (Fig. 2). Activated IFN can lead to the upregulation of several hundred IFN-stimulated genes (ISGs), which in turn promotes the secretion of several pro-inflammatory cytokines. These substances can limit viral replication and induce inflammatory responses (Ghosh et al. 2021; Guo et al. 2020; Wang et al. 2022; Zhang et al. 2019b). While inflammation serves as a crucial self-defensive mechanism, dysregulation or excessive activation of this response can result in tissue damage and the onset of inflammatory diseases. The ablation of Trex1, a gene that encodes a DNA exonuclease, results in the accumulation of fragmented DNA within the cytoplasm, consequently driving the cGAS-STING signaling pathway into a state of hyperactivation. This persistent release of pro-inflammatory cytokines and IFNs triggers an aberrant chronic inflammation (Gray et al. 2015). Considering the pivotal function of NF-κB in the regulation of pro-inflammatory gene expression such as encompassing cytokines, chemokines, and adhesion molecules, the activation of the STING-NF-κB signaling intensifies the inflammatory activity crosstalk at an additional level (Hoesel et al., 2013; Morgan et al., 2011). In a previous study, TBK1 and its homologue, IκB kinase (IKK) ε were identified as activators of the IKK complex, thereby initiating NF-κB activation (Abe et al., 2014). Interestingly, the findings of Balka et al. are different. They constructed primary macrophages lacking TBK1 and mouse models lacking TBK1, both of which demonstrated intact STING-NF-κB signaling, despite the absence of IRF3 and IFNβ. The results suggest that TBK1 and IKKε function redundantly to promote STING-induced NF-κB activation, while TAK1 and IKKβ, but not TRAF6, are essential for STING-NF-κB response (Balka et al. 2020) (Fig. 2).

### The cGAS-STING pathway and apoptosis

Apoptosis refers to the genetically orchestrated, autonomous, and systematic demise of cells (D’Arcy 2019). Recent investigations have indicated that the cGAS-STING pathway is integral to this phenomenon, significantly contributing to immune homeostasis and pathogen resistance. The interaction of IRF3 with cytoplasmic Bax through a Bcl-2 homology 3-like domain, near its carboxyl terminus, can induce the release of cytochrome c, thereby promoting apoptosis and increasing the outer mitochondrial membrane’s permeability. This alteration results in the development of pores in the outer mitochondrial membrane, allowing mtDNA to enter the cytoplasm, which further activates the cGAS-STING pathway (Chattopadhyay et al. 2010; Cui et al. 2016). Some studies showed that the production of IFN-1 modulates the expression of proteins associated with apoptosis, including members of the Bcl-2 family and BH3-only proteins, through the regulation of the JAK-STAT and PI3K-AKT signaling pathways (Cao et al. 2015; Das et al. 2016). Furthermore, STING is capable of mediating ER stress via the unfolded protein response pathway, which subsequently triggers apoptosis by the PERK and ATF6 pathways, the IRE1-TRAF2 axis, and the Caspase-12 pathway (Zheng et al. 2023).

### The cGAS-STING pathway and pyroptosis

Despite the relative independence of inflammasomes, pyroptosis, and the cGAS-STING pathway within the innate immune response, an intricate intracellular signaling network is established among these components (Liu et al. 2024). NACHT, LRR, and PYD domains-containing protein 3 (NLRP3) initiates the assembly and activation of NLRP3 inflammasomes upon the recognition of danger signals (Kelley et al., 2019). During the activation stage, Caspase-1 is recruited, triggering the release of the pro-inflammatory cytokines IL-1β and IL-18. Furthermore, Caspase-1 can cleave gasdermin D (GSDMD) to produce the GSDMD-N fragments, subsequently inducing pyroptosis (Fu et al., 2023). The crosstalk mechanisms of cGAS-STING signaling pathway, inflammasomes and pyroptosis are garnering increasing attention. In murine models of septic cardiomyopathy and diabetic cardiomyopathy, STING triggers pyroptosis via NLRP3 activation (Li et al. 2019; Yan et al. 2022). Additionally, following cGAS-STING pathway activation in human myeloid cells, STING translocates to lysosomes, resulting in lysosomal membrane destabilization. This lysosomal cell death facilitates K^+^ efflux from the cytoplasm and further activates NLRP3, consequently leading to pyroptosis (Gaidt et al. 2017).

### The cGAS-STING pathway and cellular senescence

Cellular senescence is initiated by the DNA damage response activated by various senescence-inducing stimuli, such as oxidative stress, ultraviolet radiation, and chemical agents (Regulski 2017). This phenomenon is characterized by cell cycle arrest, primarily driven by the p16INK4a/Rb and p19 ARF/p53/p21 Cip1 signaling cascades (Bielak-Zmijewska et al. 2018; He et al. 2017; Mikuła-Pietrasik et al. 2020). Additionally, it is associated with the senescence-associated secretory phenotype (SASP), which contributes to persistent inflammation and tissue dysfunction, reflecting a complex and multifaceted biological process. In certain studies, the cGAS-STING pathway is activated by remaining aberrant DNA fragments in the cytoplasm, consequently facilitating SASP production and intensifies cellular senescence (Han et al. 2020; Kang et al. 2015; Ohtani 2022; Takahashi et al. 2018; Wang et al. 2023; Wei et al., 2018). In the newly characterized cGAS-STING-PERK-eIF2α signaling cascade, STING residing on the ER activates the ER-associated kinase PERK, which precedes TBK1-IRF3 activation and is irrelevant to the unfolded protein response. Upon activation, PERK phosphorylates eIF2α, thereby facilitating selective mRNA translation that is critical for the processes of cellular senescence and the pathogenesis of fibrotic disorders (Zhang et al. 2022).

### The cGAS-STING pathway and autophagy

Autophagy is a cellular mechanism responsible for the sequestration and delivery of intracellular macromolecules and organelle constituents via autophagic vesicles to lysosomes, where they undergo catabolism and recycling (Glick et al. 2010). Classical autophagy can be initiated through the interaction between cGAS and Beclin-1, wherein this competitive binding promotes the release of the autophagy inhibitor Rubicon from the Beclin-1 complex, subsequently activating the PI3KC3 lipid kinase and enhancing autophagic activity (Zheng et al., 2021). Additionally, the ongoing recruitment of autophagy-related factors can be mediated by liquid-phase condensates formed from cGAS-dsDNA polymers (Sun et al. 2020). Despite the absence of the C-terminus in STING derived from sea anemones, it is still capable of initiating autophagy, indicating that the ability to induce autophagy might represent an evolutionarily conserved and primordial role of STING. The STING-cGAMP complex is transported from the ER to the ERGIC. STING-containing ERGIC functions as a microtubule-associated protein light chain 3 (LC3) recruiter and serves as a lipidation source that facilitates the generation of crescentic or hemicyclic autophagic precursors. cGAMP induced LC3 lipidation and subsequently triggered autophagy through a pathway that is dependent on WIPI2 and ATG5, promoting the clearance of DNA and viruses in the cytosol (Gui et al. 2019).

## The cGAS-STING pathway in CVDs: mechanisms and regulatory processes

### Atherosclerosis

Atherosclerosis is a chronic disease primarily driven by lipid accumulation and inflammatory processes (Basatemur et al. 2019; Soehnlein et al., 2021). Its progression is closely associated with endothelial cell dysfunction, inflammatory activity of macrophages, and the proliferation and migration of SMCs (Bu et al., 2023; Chistiakov et al. 2015; Geovanini et al., 2018). Endothelial cell dysfunction encompasses a constellation of various nonadaptive alterations in functional phenotype (Gimbrone et al., 2016). In the progression of smoking-induced atherosclerosis, it is reported that cigarette smoke extract induces damage to both nuclear DNA and mtDNA in endothelial cells, thereby activating the cGAS-STING-NF-κB signaling axis, which subsequently enhances the expression of pro-inflammatory cytokines such as IL-6 (Ueda et al. 2023). Additionally, the shock of low-oscillatory shear stress in blood flow has been shown to upregulate the expression of SASP genes via the cGAS-STING-NF-κB pathway, accelerating endothelial cell senescence and dysfunction (Dong et al. 2024). Several studies have further elucidated potential mechanisms of atherosclerotic endothelial cell dysfunction. An et al. found that IQ motif-containing GTPase-activating protein 1, which regulates mitochondrial function, promotes the release of mtDNA to activate the cGAS-STING-IRF3 pathway, subsequently leading to NLRP3-mediated endothelial cell pyroptosis. Notably, the application of cGAS inhibitor RU.521 or STING inhibitor C-176 reverse endothelial cells from pyroptosis (An et al. 2023). Another study of atherosclerosis focused on gasdermin E (GSDME), a protein with a role in cellular inflammation and pyroptosis. The findings demonstrate that GSDME exacerbates endothelial inflammation by impairing mtDNA, phosphorylating and activating a number of key components, including STING, TBK1, IRF3 and NF-κB (Xie et al., 2023).

Macrophages are the major immune cell population in atherosclerotic lesions and play critical roles in all stages of atherosclerosis (Barrett 2020; Colin et al. 2014; Groh et al. 2018; Tabas et al., 2016). In a mouse model of atherosclerosis, Pham et al. observed an accumulation of damaged DNA and increased STING expression in macrophages. The activation of the STING-TBK1-IRF3 and STING-NF-κB signaling cascades resulted in the elevated expression of inflammatory molecules, including TNF-α, Ccl2, and IFNβ. Importantly, the genetic deletion of STING or the use of the STING inhibitor C-176 and the TBK1 inhibitor Amlexanox, significantly decreased macrophage clustering and inflammatory responses in atherosclerotic regions (Pham et al. 2021). Additionally, research into the progression of atherosclerosis demonstrated that trans-responsive DNA-binding protein ~ 43 kDa expression was upregulated in oxidized low-density lipoprotein (oxLDL)-treated macrophages which mediated the release of mtDNA and promoted the activation of cGAS-STING-NF-κB signaling (Huangfu et al. 2021). Nevertheless, the activation of STING-NF-κB signaling can be inhibited by the natural bisbenzylisoquinoline alkaloid tetrandrine, which attenuates the inflammatory response to oxLDL attack on macrophages (Li et al. 2023b).

In the advanced phases of atherosclerosis, the proliferation of vascular smooth muscle cells (VSMCs) is crucial, which contributes to the formation and repair of the fibrous cap, thus maintaining plaque stability (Childs et al. 2021; Wang et al. 2015). A study on the vulnerability of atherosclerotic plaques has revealed that mitochondrial oxidative stress damage in VSMCs results in the release of mtDNA, which activates the cGAS-STING-TBK1-IRF3 pathway and triggers the IFN-I response. Subsequently, the enhanced IFN-1 response induces premature senescence and phenotypic transition of VSMCs in an autocrine or paracrine manner. Importantly, the aforementioned situation can be improved through the use of the STING inhibitor C-176 and its derivative H-151 (Bi et al. 2021). Notably, Uryga et al. have demonstrated that VSMC senescence contributes to neointimal formation and outward remodeling in atherosclerotic plaque intima. Telomere-derived damaged DNA has been identified as a potential activator of the cGAS-STING-NF-κB pathway. The NF-κB activates downstream SASP cytokine genes. Secreted SASP components can in turn mediate the recruitment of diverse immune and inflammatory cells to the vessel wall, which results in increased neointima formation (Uryga et al. 2021).

### MI

MI is a disease characterized by ischemic necrosis of the corresponding region of the myocardium due to obstruction of the coronary arteries (Frangogiannis 2015). Following cardiomyocyte injury resulting from insufficient oxygenation, lysate is released, causing an inflammatory response and infiltration of immune cells. Subsequently, the activation of reparative pathways promotes fibrotic scar formation and further exacerbates cardiac function (Lu et al. 2015; Toldo et al., 2018). In a mouse model of MI, the upregulation of large tumor suppressor kinase 2 has been shown to induce mitochondrial fission and the release of mtDNA, significantly enhancing the expression levels of cGAS, STING, and phosphorylated p65 in cardiomyocytes, which contribute to cardiomyocyte apoptosis (Liu et al. 2023b). King et al. demonstrated that the cGAS-STING-IRF3 pathway can be activated in macrophages by recognizing DNA from myocardial injury cells. The signaling then induces the production of IFN-1 and ISGs, which in turn amplifies post-infarction inflammation by increasing the expression of leukocyte adhesion molecules and leukocyte recruitment chemokines (King et al. 2017). The report by Cao et al. presents a perspective that the expression of ISGs triggered by the cGAS-STING pathway is a crucial factor in maintaining pro-inflammatory macrophages in MI model. Interestingly, the disruption of cGAS signaling impairs the induction of key inflammatory programs. The transition of pro-inflammatory macrophages to a reparative subtype inhibits adverse ventricular remodeling after MI (Cao et al. 2018).

The inhibition of the cGAS-STING pathway has potential to be a valuable therapeutic strategy for MI. For instance, Hu et al. utilized a STING inhibitor H-151 to treat MI mouse models. The outcomes of the study indicated that H-151 effectively suppressed the IFN-1 response and inflammation in infiltrated macrophages, which ultimately resulted in the alleviation of cardiomyocyte apoptosis and cardiac fibrosis (Hu et al. 2022). King et al. found that the knockout of cGAS, IRF3, and the IFN-1 receptor IFNAR, or the administration of IFN-1 receptor-neutralizing antibodies BioXCell, resulted in a significant increase in survival rate after MI in mice. Notably, the knock out STING or the use of the STING inhibitor C-178 did not show improved survival after MI (King et al. 2017; Rech et al. 2022).

### I/R injury

Restoring myocardial perfusion on an ischemic basis does not invariably result in the anticipated benefit. In certain instances, it can exacerbate cellular and tissue damage (Heusch 2013**)**. Following myocardial I/R injury, a variety of damage-associated molecules are released from necrotic cardiac resident cells, thus inducing an inflammatory cascade within the heart (Zhao et al. 2023). Research has indicated the involvement of the cGAS-STING signaling pathway in I/R injury. The activation of the cGAS-STING-TBK1-IRF3 signaling was observed in cardiomyocytes during both in *vivo* and in *vitro* I/R experiments, resulting in an upregulation of pro-apoptotic proteins such as Bax and Caspase-3, as well as inflammatory mediators including IL-6, IL-1β and TNFα (Xiong et al. 2023; Zhai et al. 2024). Moreover, Zhang et al. demonstrated that in ischemic and peripheral regions after I/R injury, small extracellular vesicles derived from cardiomyocyte were internalized by fibroblasts. The delivered mtDNA activated the cGAS-STING pathway. Correspondingly, pro-inflammatory cytokines were significantly upregulated, leading to myocardial fibrosis through promoting fibroblast activation and proliferation (Zhang et al. 2023).

It can be reasonably inferred that targeting the inhibition of cGAS-STING signaling may offer a novel therapeutic strategy for managing myocardial I/R injury. Li et al. discovered that scutellarin could effectively reverse the activation of the cGAS-STING pathway in I/R-injured myocardial tissue, thereby averting cardiomyocyte apoptosis. Similarly, the cGAS inhibitor RU.521 and the STING inhibitor H-151 exhibited comparable therapeutic benefits (Li et al. 2023a). The antioxidant and anti-inflammatory properties of Tanshinone IIA and Astragaloside IV were also achieved through the inhibition of STING in cardiomyocytes (Zhai et al. 2024). Furthermore, although the precise mechanisms remain unclear, the STING inhibitor C-178 has been shown to reduce infarct enlargement and scar formation following I/R injury, significantly improve left ventricular systolic function and hypertrophy (Rech et al. 2022).

### Myocarditis

Myocarditis is an inflammatory disease caused by the interaction of infectious and noninfectious factors with the immune system. The assault from various etiological factors triggers the synthesis of inflammatory cytokines and immune activation, and the persistence of the inflammatory cascade ultimately inflicts damage on cardiomyocytes, thereby facilitating the onset of cardiomyopathy (Sagar et al. 2012). Viruses, particularly Coxsackievirus B3 (CVB3), are frequently identified as the causative pathogens in instances of infectious myocarditis (Fung et al. 2016; Pollack et al. 2015). Qin et al. isolated primary cardiomyocytes from murine cardiac tissue and subsequently infected them with CVB3, demonstrating that CVB3 induces the release of mtDNA from cardiomyocytes and actives the cGAS-STING-NF-κB pathway in macrophages, which incites an inflammatory response. Similar findings were observed in a mouse model of viral myocarditis. Notably, the concentrations of inflammatory cytokines like IL-6, TNFα and MCP-1 were significantly reduced in the STING knockout group, as well as various functional indices and cardiac indexes that were closer to normal (Qin et al., 2023). In a model of experimental autoimmune myocarditis (EAM), macrophages constituted the main immune cell population at all stages of the disease, with hypoxia-inducible factor 1α (HIF1α) playing a pivotal role in promoting the pro-inflammatory polarization of macrophages (Hua et al. 2020). Further research has indicated that activated STING promoted macrophage M1 polarization through HIF1α activation, significantly influencing the progression of myocardial inflammation (Hua et al. 2023). Furthermore, in studies utilizing Trex^-/-^ mice as a model for autoimmune myocarditis, researchers observed the activation of cGAS-STING-TBK1-IRF3 pathway in bone marrow–derived macrophages, and mouse embryonic fibroblasts. The enhanced IFN-1 response induced the generation of autoantibodies and T cell activation, which are implicated in severe autoimmune inflammation (An et al. 2018; Gao et al. 2015).

The therapeutic modulation of myocarditis through the activation or inhibition of the cGAS-STING pathway has shown promise in therapeutic applications. One study demonstrated that an exosome-derived vaccine engineered with the STING agonist 2’3’ cGAMP was administered to mice with CVB3-induced viral myocarditis, resulting in enhanced CD8^+^ T cell responses and immune protection, along with a marked decrease in cardiac viral load (Zhang et al., 2024). Song et al. evaluated the effects of manassantin B (Man B) on CVB3-infected Vero cells and murine models, finding that Man B-triggered STING-TBK1-IRF3 signaling pathway suppressed CVB3 replication and improved the infection status (Song et al. 2019). Furthermore, the administration of STING inhibitor C-176, selective cGAS inhibitor RU.521, and selective HIF1α inhibitor PX-478 respectively resulted in the attenuation of the inflammatory response during the acute phase of EAM, with a significant reduction in the expression of pro-inflammatory factors in macrophages (Hua et al. 2023). A novel aminoacridine antimalarial compound X6, was found to markedly diminish the expression of cGAMP, IFN-β, and STING in the cardiac tissues of Trex^-/-^ mice, while also inhibiting endocardial inflammation and fibrosis (An et al. 2018). The aforementioned experimental results suggest the dualistic nature of STING activation, likely due to the fact that variability in the extent of STING activation significantly influence its functional outcomes; moderate STING activation appears to facilitate immune protection, whereas excessive STING activation could trigger dysregulated inflammatory responses. Consequently, it is imperative to focus on the activation threshold of STING when devising prospective immune therapeutic strategies. Alternatively, whether the inflammatory relief from STING inhibition compromises some degree of immunity remains a question to be explored.

### Cardiomyopathy

Cardiomyopathy constitutes a group of heterogeneous myocardial disorders, with etiologies that encompass various factors that leading to myocardial cellular damage, thereby resulting in alterations to cardiac architecture and functionality (McKenna et al. 2017). Cardiomyopathy can be categorized into two principal types: primary and secondary. Primary cardiomyopathy includes dilated, hypertrophic, and restrictive forms, whereas secondary cardiomyopathy arises as a consequence of underlying disease processes (Brieler et al. 2017). Dilated cardiomyopathy is the most common form of cardiomyopathy, characterized by ventricular dilation and dysfunction (Ampong 2022**)**. Notable features of dilated cardiomyopathy were observed by Chen et al. in the Lemd2 p.L13R knock-in mouse model and the corresponding cell model, which exhibited a compromised capacity for nuclear membrane repair post-rupture, leading to cytoplasmic DNA accumulation and the activation of the cGAS-STING-IFN signaling pathway. Subsequently, the pathway induced the secretion of SASP factors, contributing to the myocardial cellular senescence, apoptosis and fibrotic remodeling (Chen et al. 2023).

Diabetic cardiomyopathy represents a prevalent subtype of secondary cardiomyopathy, the pathological manifestations include lipid accumulation, mitochondrial dysfunction, inflammation, and fibrosis (Peterson et al., 2020; Tong et al. 2019). A mouse model of diabetic cardiomyopathy and a cell model (H9C2) were constructed by researchers. They observed that mitochondrial damage in cardiomyocytes resulted in the release of mtDNA into the cytoplasm, which subsequently activated the cGAS-STING-IRF3/NF-κB signaling cascade. The knockdown of STING in H9 C2 cells significantly inhibited the secretion of pro-inflammatory factors and reduced apoptosis. In the murine model, treatment with the STING inhibitor C-176 partially reversed myocardial hypertrophy, fibrosis, and dysfunction (Ma et al. 2023). Additionally, the activation of the cGAS-STING pathway was also implicated in the initiation of the NLRP3-Caspase-1-GSDMD-mediated pyroptosis program. Conversely, administration of irisin or STING silencing via adeno-associated virus 9 effectively suppressed inflammatory responses and cardiomyocyte pyroptosis (Lu et al. 2024a; Yan et al. 2022).

Various forms of secondary cardiomyopathy have garnered significant interest. The cardiotoxic effects associated with chemotherapeutic agents often limit their clinical applicability. Research has shown that cisplatin activates the cGAS-STING-TNF-α-AP-1 axis, leading to cardiomyocyte apoptosis through the upregulation of BAX expression (Wang et al. 2021). Furthermore, adriamycin has been found to promote cardiomyocyte inflammation and pyroptosis via the STING-NLRP3-Caspase-1-GSDMD signaling pathway, which can be effectively inhibited by amentoflavone (Fang et al. 2023). Chronic kidney disease (CKD) is recognized as a contributing factor to cardiac hypertrophy. Han et al. demonstrated that mitochondrial oxidative stress in cardiomyocytes, arising from CKD, stimulates the cGAS-STING-NF-κB-ornithine decarboxylase (ODC1) axis, while the use of the STING inhibitor C-176 significantly mitigates cardiac hypertrophy (Han et al. 2022). Additionally, sepsis has been found to trigger the onset of cardiomyopathy, characterized by inflammation, apoptosis, and pyroptosis (Hollenberg et al., 2021). The STING-IRF3 in cardiomyocytes has been implicated in these processes, with the mediation of NLRP3 activation. Further knockdown of STING was shown to inhibit the phosphorylation and nuclear translocation of IRF3, consequently reducing myocardial injury progression (Li et al. 2019). A murine sepsis model and a cellular model induced by LPS were employed in another investigation. Compared to the control group, the cGAS-STING-TBK1-IRF3 pathway was activated in cardiomyocytes of the experimental group, resulting in elevated levels of inflammatory mediators and reactive oxygen species, leading to exacerbated injury and apoptosis in cardiomyocytes. Notably, it has been reported that the cGAS inhibitor RU.521 and the mitochondrial aldehyde dehydrogenase 2 activator Alda-1 can mitigate the detrimental effects observed in the aforementioned studies on septic cardiomyopathy (Liu et al. 2023a; Xu et al. 2020).

### HF

HF signifies the terminal phase of various cardiovascular disorders, characterized by the heart’s inability to adequately pump blood to fulfill the metabolic requirements of the organism (McDonagh et al. 2022). Pressure overload represents a significant risk factor contributing to the onset of HF, as it alters the biomechanical properties of cardiac tissues, resulting in cardiac hypertrophy and remodeling (Liu et al. 2021). Investigations on myocardial tissues of mice challenged with angiotensin II (Ang II) or transverse aortic constriction (TAC) revealed that the inflammatory response mediated by the cGAS-STING-NF-κB axis in cardiomyocytes plays a pivotal role in the progression of HF associated with cardiac hypertrophy. Notably, the deletion of STING attenuated cardiac dysfunction in TAC mice and Ang II-induced mice (Wang et al. 2024a). Guo et al. demonstrated that pressure-induced stress in cardiomyocytes led to an upregulation of inducible nitric oxide synthase, which facilitated the release of mtDNA. This event subsequently activated the cGAS-STING-IRF3 pathway, contributing to detrimental cardiac remodeling and heart failure, whereas the knockdown of cGAS or STING alleviated these adverse effects by blunting sterile inflammation and promoting macrophage polarization towards the anti-inflammatory M2 subtype (Guo et al. 2023). Additionally, Brassington et al. elucidated the mechanisms underlying myocardial fibrosis under pressure overload, identifying NKG2D receptor-dependent CD8^+^ T cells as major contributors to cardiac fibrosis. The DNA-STING-TBK1-IRF3 signaling pathway was activated in cardiomyocytes under excessive stress, which resulted in the increased expression of the NKG2D ligand RAE-1 in conjunction with the chemokine MCP-1. Subsequently, the interaction of NKG2D and RAE-1 resulted in the activation of CD8^+^ T cells, which, in turn, induced cardiomyocyte apoptosis. Concurrently, MCP-1 attracted macrophages, and the clearance of apoptotic cells by these macrophages is essential for initiating TGF-β1 expression and myocardial fibrosis. Importantly, the aforementioned processes were effectively inhibited by the STING inhibitor H-151 (Brassington et al. 2022).

Interestingly, STING overexpression has been shown to significantly improve cardiac function. Xiong et al. observed a marked increase in mice cardiac STING protein expression in the presence of pressure overload after six weeks. These results were replicated in drug-induced cardiomyocytes and cardiac fibroblasts. The overexpression of STING demonstrated that elevated levels of STING reversed the autophagy action by promoting ULK1 phosphorylation in an AMPK/mTOR-independent manner, leading to decreased levels of autophagy-associated proteins, including Beclin-1, Atg7 and Atg12, as well as a reduced LC3II/I ratio in cardiomyocytes and fibroblasts. Consequently, the inflammatory response, cardiac hypertrophy, and fibrosis were all alleviated during cardiac remodeling (Xiong et al. 2021). It is crucial to underscore that the results pertaining to STING activation appear to display inconsistencies, necessitating a discourse. Variations in observational time points may contribute to these discrepancies; for instance, Guo et al. and Brassington et al. performed their evaluations at 3 to 4 weeks post-surgery, while Xiong et al. conducted their assessment at 6 weeks post-surgery. Furthermore, the pathology in the early stages of HF models may be characterized by the dysregulation of inflammatory responses, wherein the recruitment of immune cells exacerbates the STING-mediated inflammatory cascades. As the disease advances, the decline in immune cell populations permits the function of STING in autophagy modulation to progressively assume prominence.

### AAD

The impairment of the structural and functional integrity of aortic components precipitates aortic degeneration, characterized by the progressive depletion of VSMCs and the degradation of the extracellular matrix (Shen et al. 2020). Persistent vascular inflammation has been identified as a contributor to the pathogenesis of AAD (Okrzeja et al., 2022; Postnov et al. 2021; Yuan et al. 2020). Luo et al. found that in human sporadic ascending thoracic AAD tissues, the cytoplasmic DNA levels were significantly elevated compared to controls, alongside marked upregulation of STING, TBK1, and IRF3 expression and phosphorylation. Further investigation revealed that stress responses, such as reactive oxygen species, triggered DNA damage and activated the cGAS-STING-TBK1-IRF3 pathway in aortic SMCs resulting in necrosis and apoptosis of SMCs. DNA released from damaged or dead SMCs further activates the STING-IRF3 signaling pathway in macrophages, leading to matrix metalloproteinase (MMP)−9 production and cytoplasmic matrix degradation (Luo et al. 2020). Chakraborty et al. conducted a detailed study on the phenotypic transformation of SMCs in AAD. Through single-cell RNA sequencing analysis and validation experiments, it was revealed that dsDNA-STING-TBK1-IRF3 signaling reduces the expression of SMCs contractile genes while promoting the expression of inflammatory genes by recruiting enhancer of zeste homolog 2 and inducing repressive H3 K27 me3 modifications and chromatin remodeling. This mechanism facilitates the transformation of SMCs from a well-differentiated contractile phenotype to a pro-inflammatory and pro-death phenotype, ultimately leading to aortic degeneration and dysfunction. Notably, while a decrease in the population of SMCs was observed in the STING-deficient AAD mouse model, the proportion remained elevated compared to unstressed aortas, indicating that additional pathways may similarly contribute to the inflammatory transition of SMCs (Chakraborty et al. 2023).

The cGAS-STING pathway has potential to be a new therapeutic target for AAD. The TBK1 inhibitor Amlexanox was applied to SMCs, resulting in the partial inhibition of STING and IRF3 phosphorylation, as well as a decrease in SMC apoptosis and activation of macrophages. Following the infusion of Amlexanox and a control agent into the mouse models of sporadic AAD, the Amlexanox-treated group showed better retention of aortic structure, reduced morbidity and severity, and enhanced survival rates (Luo et al. 2020). Furthermore, STING deficiency has been found to reduced SMC adverse phenotypic alterations and preserved the SMC population (Chakraborty et al. 2023). These results underscore the potential of targeting STING as a promising strategy.

## The modulators of the cGAS-STING pathway in CVDs

Considering the critical role of the cGAS-STING signaling axis in the pathophysiology of cardiovascular disorders, it is plausible to target this pathway to mitigate inflammatory responses, cellular senescence, and cell death as a therapeutic approach. Next, we encapsulate some initiatives and endeavors that have been undertaken (Table 2).

### cGAS inhibitors

The effects of cGAS inhibitors typically include (1) inhibition of the enzymatic activity of cGAS (Lama et al. 2019). Competitive inhibitor RU.521 can bind to the active site of cGAS, obstructing the catalytic production of cGAMP by inhibiting its interaction with ATP and GTP (Vincent et al. 2017). Additionally, substrate-competitive inhibitors PF-06928215 directly impede cGAS’s catalytic activity, alleviating palmitate-induced contractile impairment in cardiomyocytes (Hall et al. 2017; Xu et al. 2020). (2) Disruption of cGAS binding to DNA, such as X6, a series of antimalarial drugs bearing aminoacridine or aminoquinolone, Suramin, and A151, these compounds prevent the formation of the cGAS-dsDNA complex by binding to the DNA groove at the cGAS/DNA interface (An et al. 2018; An et al. 2015; Ehsanian et al. 2011; Steinhagen et al. 2018; Wang et al. 2018). (3) Targeted modulation of cGAS. Recent investigations have indicated that deacetylation of cGAS upon DNA treatment is involved in cGAS activation. The commonly used non-steroidal anti-inflammatory drug aspirin can inhibit self-DNA-mediated autoimmune responses in Aicardi-Goutières syndrome (AGS) patient cells and AGS mouse model by obligatory acetylation of Lys384, Lys394 or Lys414 on cGAS (Dai et al. 2019). In conclusion, cGAS inhibitors have exhibited considerable therapeutic efficacy in certain disease models and may hold clinical significance for the intervention of CVDs.

### STING inhibitors and agonists

The primary mechanisms through which STING inhibitors exert their effects involve the disruption of the palmitoylation pathway and the competitive blockade of STING’s interaction with cyclic dinucleotides (CDNs) (Ding et al. 2020). Through screening and characterization, the nitrofuran derivatives C-176, C-178, and H-151 were found to effectively inhibit the palmitoylation of STING by binding to Cys91 on the STING protein (Haag et al. 2018). Nitro fatty acids can attenuate STING signaling through the nitroalkylation of STING’s palmitoylation site, offering therapeutic relief in STING-mediated inflammatory disorders (Hansen et al. 2018; Mollenhauer et al. 2020). Generally, the interaction of CDNs with CDN-binding domain on STING promotes a conformational transition from the “open” to the “closed” state within its dimeric configuration, thereby initiating downstream signaling cascades. Compounds AstinC and SN-011 act as competitors to CDNs by occupying the catalytic activation pocket of STING (Hong et al., 2021; Li et al. 2018). Tetrahydroisoquinolone, derived through platform recognition, demonstrates the ability to bind to the STING homodimer in a 2:1 ratio, subsequently stabilizing its “open” conformation. The inactivation of STING impeding the signaling processes (Siu et al. 2019).

Notably, the stimulation of the STING-IRF3 signaling pathway for the regulating of IFN induction is increasingly recognized as a viable strategy for establishing an immunogenic environment (Liang et al. 2023; Song et al. 2019). Naturally CDNs, such as 2′3′ cGAMP and its three isomeri forms distinguished by their phosphodiester linkages, frequently serve as direct STING agonists (Zhang et al. 2013). Nonetheless, the clinical use of these compounds is restricted due to their vulnerability to hydrolysis by phosphodiesterases and ineffective intracellular delivery (Wu et al. 2020). Altering and optimizing the architecture of natural CDNs represents a significant avenue for future development. A range of synthetic CDNs derivatives, including ADU-S100 and cyclic adenosine-inosine monophosphates (cAIMPs), have been demonstrated enhanced stability and potency (Corrales et al. 2015; Lioux et al. 2016). An alternative strategy involves employing liposomal carriers or nanomultimers, for the encapsulation of CDNs, thus optimizing their cellular membrane permeability and addressing the challenges associated with low response rates (Xu et al., 2024). Overall, the advancement of STING inhibitors and agonists is anticipated to confer a protective effect in CVDs.

### TBK1 inhibitors

The identification of TBK1 inhibitors presents promising therapeutic avenues for managing related diseases. Initially, 2,4-Diaminopyrimidine (BX795) was designed as a specific inhibitor of 3- phosphoinositide-dependent protein kinase 1, yet it has shown notable efficacy against a variety of other kinases. The aminopyrimidine core establishes a hydrogen bond network within the hinge-binding region of the kinase, thereby obstructing the autophosphorylation of TBK1 and IKKε at the Ser172 site (Clark et al. 2009). Consequently, nearly all early inhibitors incorporated a central aminopyrimidine structure (Clark et al. 2011). Subsequent activity-based screenings of extensive compound libraries have uncovered several kinase inhibitors, including K252a, dovitinib, oxindole 91, and additional aminopyrimidines, which exhibit a mechanism of action akin to that of BX795 (Richters et al. 2015). Presently, Amlexanox, a medication utilized for treating ulcers and asthma, has been observed to inhibit TBK1 phosphorylation at Ser366, effectively mitigating the negative effects of STING activation in mouse models of atherosclerosis and AAD (Luo et al. 2020; Pham et al. 2021; Zhou et al. 2020).

### Botanical extracts

“Enhancing qi and promoting blood circulation” represents a significant aspect of the therapeutic strategies developed within traditional Chinese medicine for addressing cardiovascular ailments (Li et al. 2020). The primary bioactive components, Astragaloside IV and Tanshinone IIA, derived from Astragalus membranaceus, a recognized qi tonic, and Salvia miltiorrhiza, a noted blood-activating agent, can be utilized either separately or synergistically to exert anti-inflammatory and antioxidant actions via inhibiting STING phosphorylation (Zhai et al. 2024). Another prominent Chinese herbal formulation, Compound Danshen Dropping Pill (CDDP), primarily consists of Danshen and Panax ginseng. Mechanistically, CDDP remarkably eliminated the STING-TBK1 interaction and its use has shown efficacy in mitigating inflammatory and autoimmune diseases (Shi et al. 2024). Various natural extracts, including Amentoflavone from Ginkgo biloba, St. John’s wort and other plants, flavonoid from Epimedium, and Tetrandrine, a bisbenzylisoquinoline alkaloid sourced from the roots of Stephania tetrandra, have been demonstrated the ability to obstruct the assembly of functional STING signalosomes, potentially offering therapeutic benefits for inflammatory disorders (Fang et al. 2023; Li et al. 2023b; Wang et al. 2024b). Furthermore, Liu et al. demonstrated that epigallocatechin gallate, a polyphenolic compound derived from tea leaves, specifically attenuates cGAS-dependent IFN production in AGS patient cells and AGS mouse model by disrupting the interaction between G3BP1 and cGAS (Liu et al. 2019).

### Endogenous compounds

The synthetic compounds present in biological organisms are key factors to the intricate regulation, repair mechanisms, and environmental adaptation of the body (Hoffmann et al. 2023; Kraemer et al., 2017). Following physical activity, skeletal muscles enhance the secretion of irisin to various tissues, which subsequently ameliorates or rectifies a state of energy metabolism imbalance (Bao et al. 2022). Lu et al. demonstrated that irisin supplementation markedly elevated the expression of the mitochondrial ubiquitin ligase MITOL and maintained mitochondrial function in a model of diabetic cardiomyopathy, which was accompanied by decreased activation of the cGAS-STING pathway and GSDMD-dependent pyroptosis (Lu et al. 2024a). Liu et al. found that the increased mitochondrial aldehyde dehydrogenase 2 alleviated LPS-induced cytotoxicity by reducing mitochondrial membrane permeability and reversed inflammatory responses and apoptosis induced by the cGAS-STING signaling (Liu et al. 2023a). Furthermore, the elevation of Meteorin-like hormone (Metrnl), a secreted protein widely distributed in the body, mitigates STING-mediated cardiac hypertrophy and fibrosis. Metrnl promoted ULK1 phosphorylation by enhancing autophagic flux, thereby inhibiting cGAS-STING signaling in cardiomyocytes. Alternatively, Metrnl facilitated the ubiquitination-dependent degradation of STING by fostering the formation of a TRAF2/STING complex within the mitochondria of cardiomyocytes (Lu et al. 2023).

## Conclusions and future perspectives

This review provides a summary of the structural features of cGAS and STING that are crucial for their biological roles. In the progression of CVDs, the cGAS-STING pathway is primarily activated by mtDNA and functions at the cellular level, often manifesting as promotion of cellular inflammatory responses, cellular senescence and various forms of cell death (Table 1). The inhibition or activation of the cGAS-STING pathway has been observed to reduce cardiac inflammation and injury, indicating that the cGAS-STING pathway has potential to be a novel target for CVDs therapy. Present preclinical investigations are concentrating on the targeting mechanisms at distinct stages of cGAS-STING signaling activation. Additionally, an expanding array of natural products and endogenous factors has been identified as potential CVD treatments through pharmacological actions linked to cGAS-STING (Fang et al. 2023; Zhai et al. 2024) (Table 2). While animal model studies underscore the significance of the cGAS-STING signaling axis in the modulation of cardiovascular pathologies, the implementation of targeted therapeutics aimed at cGAS-STING encounters numerous hurdles in clinical settings. These challenges may stem from the off-target effects of small molecule inhibitors and concerns regarding their long-term safety profiles. Subsequent research endeavors should concentrate on enhancing the specificity of cGAS-STING inhibitors, potentially leveraging innovative methodologies such as computer-aided drug design to refine their targeting and mitigate adverse effects. Furthermore, considering that DNA leakage is an antecedent to the activation of the cGAS-STING pathway, forthcoming clinical studies could focus on evaluating the correlation between CVDs and plasma free DNA levels, particularly the potential of mitochondrial cell-free DNA as a predictive biomarker for cardiovascular events. 

**Table 1:** Overview of the cGAS-STING pathway in cardiovascular diseases

The regulatory mechanisms	Cell types	Activators	Effects	Therapeutic interventions	Reference
**Atherosclerosis**					
1.cGAS-STING-TBK1-IRF3	EC	mtDNA	Inflammatory responses↑		(Xie et al., 2023)
			Pyroptosis↑	cGAS inhibitor RU.521 STING inhibitor C-176	(An et al. 2023)
	Macrophage	mtDNA	Inflammatory responses↑	STING inhibitor C-176	(Pham et al. 2021)
	SMC	mtDNA	Cellular senescence and phenotypic transition↑	STING inhibitor C-176 STING inhibitor H-151	(Bi et al. 2021)
2.cGAS-STING-NF-κB	EC	mtDNA, nuclear DNA	Inflammatory responses↑		(Ueda et al. 2023)
			Cellular senescence↑	STING inhibitor C-176	(Dong et al. 2024)
	Macrophage	mtDNA	Inflammatory responses↑	STING inhibitor C-176 TBK1 inhibitor Amlexanox Tetrandrine	(Huangfu et al. 2021; Li et al. 2023b; Pham et al. 2021)
	SMC	Telomere DNA	Inflammatory responses↑ Secretion of SASP↑		(Uryga et al. 2021)
**Myocardial infarction**					
1.cGAS-STING-TBK1-IRF3	Macrophage	Cardiomyocyte DNA	Inflammatory responses↑	STING inhibitor H-151 IFN-1 receptor-neutralizing antibodies BioXCell	(Hu et al. 2022; King et al. 2017)
			Maintained M1 macrophage polarization		(Cao et al. 2018)
2.cGAS-STING-NF-κB	Cardiomyocyte	mtDNA	Apoptosis↑		(Liu et al. 2023b)
**Ischemia-reperfusion injury**					
1.cGAS-STING-TBK1-IRF3	Cardiomyocyte	mtDNA	Inflammatory responses↑ Apoptosis↑	cGAS inhibitor RU.521 STING inhibitor H-151 Tanshinone IIA Astragaloside IV Scutellarin	(Li et al. 2023a; Xiong et al. 2023; Zhai et al. 2024)
**Myocarditis**					
1.cGAS-STING-TBK1-IRF3	Macrophage	Cytosolic DNA	Inflammatory responses↑ Autoantibody production↑	cGAS inhibitor X6	(An et al. 2018; Gao et al. 2015)
2.cGAS-STING-NF-κB	Macrophage	mtDNA	Inflammatory responses↑		(Qin et al., 2023)
3.cGAS-STING-HIF1α	Macrophage	mtDNA	Inflammatory responses↑ M1 macrophage polarization↑	cGAS inhibitor RU.521 STING inhibitor C-176 HIF1α inhibitor PX-478	(Hua et al. 2023)
**Cardiomyopathy**					
1.cGAS-STING-TBK1-IRF3	Cardiomyocyte	mtDNA	Apoptosis↑	cGAS inhibitor RU.521 STING inhibitor C-176 Aldehyde dehydrogenase 2 activator Alda-1	(Liu et al. 2023a; Ma et al. 2023; Xu et al. 2020)
			Pyroptosis↑	Irisin Amentoflavone	(Fang et al. 2023; Li et al. 2019; Lu et al. 2024a; Yan et al. 2022)
		Nuclear DNA	Cellular senescence↑		(Chen et al. 2023)
2.cGAS-STING-NF-κB	Cardiomyocyte	mtDNA	Inflammatory responses↑ Hypertrophy↑	STING inhibitor C-176	(Han et al. 2022; Ma et al. 2023)
3.cGAS-STING-TNF-α-AP-1	Cardiomyocyte	mtDNA	Apoptosis↑		(Wang et al. 2021)
**Heart failure**					
1.cGAS-STING-TBK1-IRF3	Cardiomyocyte	mtDNA	Inflammatory responses↑		(Guo et al. 2023)
			Apoptosis↑	STING inhibitor H-151	(Brassington et al. 2022)
2.cGAS-STING-NF-κB	Cardiomyocyte	mtDNA	Inflammatory responses↑	STING inhibitor H-151	(Wang et al. 2024a)
**Aortic aneurysm and dissection**					
1.cGAS-STING-TBK1-IRF3	SMC	mtDNA, nuclear DNA	Necrosis and apoptosis↑	TBK1 inhibitor Amlexanox	(Luo et al. 2020)
		mtDNA	Pro-inflammatory and pro-death phenotype↑		(Chakraborty et al. 2023)
	Macrophage	SMC DNA	Cytoplasmic matrix degradation↑ MMP-9 production↑	TBK1 inhibitor Amlexanox	(Luo et al. 2020)

*EC* endothelial cell; *SMC* smooth muscle cell; *MMP-9* matrix metalloproteinase-9

**Table 2:** Overview of the cGAS-STING pathway modulators in cardiovascular diseases

Roles	Molecular mechanisms	Names	Disease models	Reference
cGAS inhibitors	Inhibiting the enzymatic activity of cGAS	RU.521	AS, I/R injury, SCM, EAM	(An et al. 2023; Hua et al. 2023; Li et al. 2023a; Xu et al. 2020)
		PF-06928215	Cardiac anomalies	(Gong et al. 2020)
	Disrupting cGAS binding to DNA	X6	EAM	(An et al. 2018)
		Hydroxychloroquine		(An et al. 2015)
		Quinacrine		(Ehsanian et al. 2011)
		Suramin		(Wang et al. 2018)
		A151	AD	(Steinhagen et al. 2018)
	Acetylating three lysine residues of cGAS	Aspirin	AD	(Dai et al. 2019)
STING inhibitors	Inhibiting palmitoylation site of STING	C-176	AS, EAM, DCM, CKD	(An et al. 2023; Han et al. 2022; Hua et al. 2023; Pham et al. 2021)
		C-178	I/R injury	(Rech et al. 2022)
		H-151	AS, MI, I/R injury, HF	(Bi et al. 2021; Brassington et al. 2022; Hu et al. 2022; Li et al. 2023a)
		Nitro-fatty acids		(Hansen et al. 2018)
	Blocking CDNs in combination with STING	AstinC	AD	(Li et al. 2018)
		Tetrahydroisoquinolone		(Siu et al. 2019)
		SN-011	AD	(Hong et al., 2021)
STING agonists	CDNs	2′2′ cGAMP		(Zhang et al. 2013)
		2′3′ cGAMP	VM	(Zhang et al., 2024)
		3′2′ cGAMP		(Zhang et al. 2013)
		3′3′ cGAMP		(Zhang et al. 2013)
	CDNs derivatives	ADU-S100		(Corrales et al. 2015)
		cAIMPs		(Lioux et al. 2016)
TBK1 inhibitors	Inhibiting TBK1-induced phosphorylation	BX795		(Clark et al. 2009)
		MRT67307		(Clark et al. 2011)
		K252a		(Richters et al. 2015)
		Dovitinib		(Richters et al. 2015)
		Oxindole 91		(Richters et al. 2015)
		Amlexanox	AS, AAD	(Luo et al. 2020; Pham et al. 2021)
Botanical extracts	Inhibiting STING phosphorylation	Tanshinone IIA	I/R injury	(Zhai et al. 2024)
		Astragaloside IV	I/R injury	(Zhai et al. 2024)
		Amentoflavone	Cardiotoxicity	(Fang et al. 2023)
	Inhibiting TBK1 phosphorylation	Tetrandrine	AS	(Li et al. 2023b)
	Inhibiting STING-TBK1 interaction	Compound Danshen Dripping Pill	AD	(Shi et al. 2024)
	Inhibiting the interaction of IRF3 with STING and TBK1	Epimedium flavonoid	AD	(Wang et al. 2024b)
	Disrupting the interaction between G3BP1 and cGAS	Epigallocatechin gallate	AD	(Liu et al. 2019)
Endogenous compounds	Reducing mitochondrial damage	Irisin	DCM	(Lu et al. 2024a)
		Aldehyde dehydrogenase 2	SCM	(Liu et al. 2023a)
	Promoting dephosphorylation of STING	Metrnl	DCM	(Lu et al. 2023)

*AS* atherosclerosis; *I/R* ischemia-reperfusion; *SCM* septic cardiomyopathy; *EAM* experimental autoimmune myocarditis; *AD* autoimmune disease; *DCM* diabetic cardiomyopathy; *CKD* chronic kidney disease; *MI* myocardial infarction; *HF* heart failure; *AAD* aortic aneurysm and dissection; *VM* viral myocarditis

Notably, preclinical studies indicate that the application of cGAS-STING pathway modulators may elicit adverse effects, such as the induction of dysregulated inflammation or compromised degree of immunity. Consequently, it is imperative for investigators to ensure that subjects in clinical trials are thoroughly informed of the potential benefits and risks to prevent ethical dilemmas in translational research.

## Data Availability

No datasets were generated or analysed during the current study.

## Abbreviations

CVDs: Cardiovascular diseases
HF: Heart failure
cGAMP: Cyclic GMP-AMP
cGAS: Cyclic GMP-AMP synthase
STING: Stimulator of interferon gene
dsDNA: Double-stranded DNA
TBK1: TANK-binding kinase 1
IRF3: Interferon regulatory factor 3
IFN: Interferon
IFN-1: Type I interferon
IKK: IκB kinase
NF-κB: Nuclear factor kappa-B
MI: Myocardial infarction
I/R: Ischemia-reperfusion
AAD: Aortic aneurysm and dissection
SMCs: Smooth muscle cells
NTase: Nucleotidyltransferase
ATP: Adenosine triphosphate
GTP: Guanosine triphosphate
CTD: C-terminal structural domain
TM: Transmembrane
LBD: Ligand-binding domain
ER: Endoplasmic reticulum
CTT: C-terminal tail
ERGIC: ER–Golgi intermediate compartment
TBM: TBK1 binding motif
ISGs: IFN-stimulated genes
mtDNA: Mitochondrial DNA
TFAM: Mitochondrial transcription factor A
NLRP3: NACHT, LRR, and PYD domains-containing protein 3
GSDMD: Gasdermin D
SASP: Senescence-associated secretory phenotype
LC3: Light chain 3
GSDME: Gasdermin E
oxLDL: Oxidized low-density lipoprotein
VSMCs: Vascular smooth muscle cells
CVB3: Coxsackievirus B3
EAM: Experimental autoimmune myocarditis
HIF1α: Hypoxia-inducible factor 1α
Man B: Manassantin B
LPS: Lipopolysaccharide
CKD: Chronic kidney disease
ODC1: Ornithine decarboxylase
Ang II: Angiotensin II
TAC: Transverse aortic constriction
MMP: Matrix metalloproteinase
AGS: Aicardi-Goutières syndrome
CDNs: Cyclic dinucleotides
cAIMPs: Cyclic adenosine-inosine monophosphates
CDDP: Compound Danshen Dropping Pill
Metrnl: Meteorin-like hormone
